# Enhanced Dual-Selection Krill Herd Strategy for Optimizing Network Lifetime and Stability in Wireless Sensor Networks

**DOI:** 10.3390/s23177485

**Published:** 2023-08-28

**Authors:** Allam Balaram, Rajendiran Babu, Miroslav Mahdal, Dowlath Fathima, Neeraj Panwar, Janjhyam Venkata Naga Ramesh, Muniyandy Elangovan

**Affiliations:** 1 Department of Computer Science and Engineering, MLR Institute of Technology, Hyderabad 500043, India; drbalaramallam@mlrinstitutions.ac.in; 2 Department of Computational Intelligence, School of Computing, SRM Institute of Science and Technology, Chennai 603203, India; babur@srmist.edu.in; 3 Department of Control Systems and Instrumentation, Faculty of Mechanical Engineering, VSB-Technical University of Ostrava, 17. Listopadu 2172/15, 70800 Ostrava, Czech Republic; 4 Department of Basic Sciences, College of Science and Theoretical Studies, Saudi Electronic University, Riyadh 11673, Saudi Arabia; d.fathima@seu.edu.sa; 5 School of Computing, Graphic Era Hill University, Dehradun 248002, India; panwar@gehu.ac.in; 6 Department of Computer Science and Engineering, Koneru Lakshmaiah Education Foundation, Guntur 522302, India; jvnramesh@kluniversity.in; 7 Department of Biosciences, Saveetha School of Engineering, Saveetha Nagar, Thandalam 602105, India; drmelangovan@veltech.edu.in; 8 Department of R&D, Bond Marine Consultancy, London EC1V 2NX, UK

**Keywords:** krill herd, dual mechanism, stability, latency, exploration, exploitation

## Abstract

Wireless sensor networks (WSNs) enable communication among sensor nodes and require efficient energy management for optimal operation under various conditions. Key challenges include maximizing network lifetime, coverage area, and effective data aggregation and planning. A longer network lifetime contributes to improved data transfer durability, sensor conservation, and scalability. In this paper, an enhanced dual-selection krill herd (KH) optimization clustering scheme for resource-efficient WSNs with minimal overhead is introduced. The proposed approach increases overall energy utilization and reduces inter-node communication, addressing energy conservation challenges in node deployment and clustering for WSNs as optimization problems. A dynamic layering mechanism is employed to prevent repetitive selection of the same cluster head nodes, ensuring effective dual selection. Our algorithm is designed to identify the optimal solution through enhanced exploitation and exploration processes, leveraging a modified krill-based clustering method. Comparative analysis with benchmark approaches demonstrates that the proposed model enhances network lifetime by 23.21%, increases stable energy by 19.84%, and reduces network latency by 22.88%, offering a more efficient and reliable solution for WSN energy management.

## 1. Introduction

Due to their suitability and utility in a range of circumstances, such as military operations, surveillance, health monitoring, weather forecasting, and other applications, wireless sensor networks (WSNs) are expected to be omnipresent in human daily life [1,2]. A WSN is made up of millions of sensor nodes to help with sensing and data aggregation operations [3,4]. The essential challenges of processing time, limited energy, and constrained memory and capability, however, are influenced by the size of the sensor nodes [5,6]. In this scenario, the network’s longevity is dependent on the number of resources that are available. The balance of network resources is also influenced by the choice of an appropriate clustering algorithm for use in routing processes [7,8]. Currently, the challenging problem is determining how to effectively manage both the network as a whole and the clusters of closely spaced sensor nodes for adaptation [9,10]. Their potential is determined by the cluster head assortment procedure included in the clustering algorithms [11,12]. Moreover, the clustering algorithms must successfully maintain the network’s sensor nodes’ energy balance [13,14]. With an increase in network longevity as their main objective, the majority of energy-balancing clustering algorithms presented lately are either probabilistic or random in nature [15,16]. When the sensor nodes closest towards the base station use up the majority of their energy earlier than the nodes farther away, it is discovered that cluster head or random selection techniques are less successful [17,18]. Moreover, random cluster head selection procedures contribute to the hotspot issue. Sensor nodes die earlier as a result of hotspot issues, dramatically lowering network performance [19,20]. On top of that, the most recent probabilistic cluster head selection methods [21,22] only considered the sensor nodes’ prior rotating experience. Moreover, it is thought that choosing the cluster head is an NP-complete problem.

### 1.1. Challenges Dealt with in WSNs

The lifespan of the network is therefore believed to be greatly increased by the selection of cluster heads utilizing potential meta-heuristic procedures [23]. Metaheuristics find wide application in various fields [24,25,26,27,28]. For cluster head schemes, a number of algorithms have been presented in the literature, including the ant colony optimization, harmony search algorithm, elephant herd optimization, artificial bee colony, cuckoo search algorithm, and particle swarm optimization algorithms. When utilized as standalone algorithms, the proposed methods do not, however, appreciably enhance cluster head selection performance [29,30]. Due to the use of hybrid and altered EHO algorithms for cluster head selection operations, the standalone EHO algorithms have undergone a few changes that have garnered more interest [31,32]. Due to the fundamental properties of hybridized and modified EHO, a new selection technique was created in order to prolong the network lifetime while maximizing energy conservation. It is suggested that the clustering method will make the energy-balanced cluster head selection simpler and increase the lifetime of the network [33,34].

### 1.2. Proposed Idea along with Its Novelty

The proposed EDS strategy, which strengthened the elitism and prevented the cluster leader from being chosen from the worst sensor node, was extended with six essential updating mechanisms [35,36]. It also utilized the advantages of clan and separation operators to accomplish local and global search goals over the entire population’s search space. End-to-end mean latency, mean packet delivery rate, and mean residual energy under half network lifespan under different network sizes are used in the simulation studies of the recommended EDS-KH method [37,38].

The primary advantages of the suggested scheme are as follows:

- It is suggested to find and select a cluster head with an extraordinary balance in the rate of exploitation, which aids in better clustering and extends the life of the network.
- As part of the elephant herd optimization process, a variety of exploration strategies are used for a significant improvement in energy conservation and network efficiency.
- The separation and clan operator, which is essential for selecting cluster heads successfully to prevent energy leaks in the network, is updated and passed down to it.
- It has a fitness function that includes a maximum number of components, which is crucial for choosing dominant cluster heads and protecting against network deterioration.

The remaining sections of the article are as follows: In Section 2 of this study, in-depth coverage is given of the key modern swarm-intelligence cluster head selection methods. The entire description of the suggested method is provided in Section 3 together with the pertinent justifications. Section 4 focuses on the results and discussion of the planned EDS-KH system, in addition to the reasons for its successful implementation. In Section 5, there is a review of the paper, which highlights the substantial contributions of the proposed work in light of prospective advancements in the future.

## 2. Related Works

The main contributions of the literature that provided the framework and inspiration for the creation of the suggested system are discussed in this section along with their benefits and drawbacks.

To show how a sampling technique may be utilized to select the ideal cluster head, [30] suggested a sampling-based spider monkey optimization-based clustering technique (SSMO-CS). Due to the sampling concept, it prohibited the multiple selections of sensor nodes as cluster heads. The best samples allowed for the best results, even if sampling was only relevant to the search process. The SSMO-CS simulation testing ultimately demonstrated its superiority in terms of energy adaptability. Then, using a finite state machine, ref. [39] planned the clustering strategy (FSM). The Marko model was employed in this FSM-rooted cluster head selection technique to foretell the cluster selection around the subsequent state. It concentrated on choosing the best cluster head relying on the current energy consumption and estimated distance factor of the sensor nodes belonging to the network. By increasing lifespan and throughput over LEACH and C-LEACH systems by 1.35 times and 1.12 times, respectively, it showed how significant the node-scheduling-based cluster head selection technique is.

Furthermore, utilizing the fractional lion algorithm, ref. [40] proposed a CHSS-FLA (cluster head selection method). The fractional lion algorithm (FLA) was used to construct a routing scheme that was energy-efficient. It increased energy and extended longevity by quickly choosing cluster heads. The CHSS-FLA fitness function was developed using factors such as the cluster head’s energy, the sensor nodes’ energy, the delay, and the distances inside and between clusters for the sensors and the base station. With regard to network endurance and stored residual energy, the CHSS-FLA-simulated simulations demonstrated its pervasiveness. To prolong the life of the network, for the purpose of choosing the cluster head, ref. [41] proposed the LEACH-IBA, an integrated LEACH and optimized bat algorithm. This LEACH-IBA made use of the curve technique to broaden the scope of abilities pertaining to local and global searches. To keep the clustering process active, it makes use of the benefits of the LEACH plus bat algorithms. Since LEACH-IBA boosts throughput and network longevity by 2.38 and 2.12 times, respectively, compared to ABC-based clustering algorithms, it outperforms them.

Moreover, ref. [42] developed a hybrid cluster head selection method relying on the fruit fly and glowworm swarm algorithms (HCSA-FGSA). After a thorough investigation of energy and live nodes, it was discovered that the cost function was significantly minimized and comparable to the present clustering algorithms. Ref. [43] used an altered ACO (IABC-ACO-OCHS) and ABC to remove frequent cluster heads, which in turn raised the energy stability overhead during the routing procedure. It is found that the ABC-ACO-OCHS scheme significantly makes it easier to maintain the mean delay and residual energy, regardless of the network size, in a flexible manner.

Moreover, ref. [44] suggested a merged MBO- and ABC-based clustering technique that is crucial for lengthening the lifetime by matching the costs of global and local searches. The main component causing the convergence of the search process, the butterfly adjustment factor, has been shown to be a useful tool for controlling the speed of both international and domestic searches. The gap between exploitation and exploration was bridged in [45] with the development of a method known as optimum cluster head selection with the krill herd algorithm. Independent of network size, it has also been demonstrated that the KHA-OCHS scheme’s residual energy and mean latency are remarkably robust and flexible.

### Problems Identified

Wireless sensor networks (WSNs) face an array of formidable challenges, encompassing limited processing time, constrained energy resources, and restricted memory and computational capabilities. The computational constraints of sensor nodes often lead to sluggish data processing, introducing delays in data transmission and decision-making processes and thereby adversely impacting the overall network efficiency. Moreover, the finite energy reservoirs of sensor nodes raise concerns about the network’s longevity, as excessive energy consumption can precipitate premature battery depletion, rendering nodes non-operational and disrupting network operations. Furthermore, the constrained memory and capability curtail the execution of intricate tasks and data processing, restricting the node’s potential for sophisticated operations. To overcome these challenges and ensure the sustainable performance of WSNs, the development of innovative algorithms, data aggregation techniques, and intelligent routing protocols becomes imperative for optimizing energy utilization while maximizing the network’s lifespan and efficacy. Embracing advanced power management and energy harvesting solutions can further fortify the WSNs, fostering resilience and elevating their potential for diverse application domains.

This study seeks to explore the following research questions and objectives:

Research Question 1: Can we design a clustering algorithm that dynamically adapts to changing network conditions and optimizes the selection of cluster heads?

Objective 1: Develop a novel clustering algorithm that utilizes adaptive mechanisms to dynamically adjust cluster head selection based on changing network parameters and node conditions.

Research Question 2: How can we achieve energy balance and prolong the network’s lifetime through improved cluster head selection?

Objective 2: Integrate energy-balancing techniques into the clustering algorithm to ensure fair distribution of energy consumption among sensor nodes, thereby prolonging the network’s lifetime.

Research Question 3: Can the proposed algorithm efficiently handle the challenges posed by irregularly shaped clusters and varying cluster densities?

Objective 3: Enhance the clustering algorithm to handle non-spherical and overlapping clusters, as well as varying densities, to improve cluster formation accuracy and data aggregation efficiency.

Research Question 4: What is the impact of the proposed algorithm on network scalability and data aggregation efficiency?

Objective 4: Evaluate the scalability of the proposed algorithm in large-scale WSNs and assess its performance in terms of data aggregation and fusion to reduce communication overhead.

Research Question 5: Does the new clustering algorithm enhance fault tolerance and network robustness?

Objective 5: Investigate the impact of the proposed algorithm on the network’s fault tolerance and robustness, ensuring the network can withstand node failures and environmental changes.

By addressing these research questions and achieving the defined objectives, the study aims to contribute to the field of WSNs by proposing a clustering algorithm that is adaptive, energy-efficient, scalable, and robust. 

## 3. Proposed Methodology for Effectual Cluster Head Selection

In this section, the selection of the CH that uses the GA and the KH is discussed.

### 3.1. CH Selection Using Genetic Algorithm

John Holland first presented the GA in 1970, based on the principle of evolution as outlined by Charles Darwin. This adaptive heuristic technique is used to resolve dynamic issues and is based on genetic evolution. This algorithm was employed for solving several other NP-hard problems, but encoding a problem regarding a particular set of chromosomes in which each chromosome is a clarification is a primary issue in solving a problem with GA. To gauge the chromosomal quality, a fitness function is employed. Operations of crossover and mutation are used in accordance with the fitness value depending on the chosen chromosomes. By means of the concatenation of the elements of both selected chromosomes, new solutions called offspring are generated. For all offspring produced, a mutation is used to change one or more of the genetic elements to avoid the solution from being trapped inside local minima. The recommended CH selection process solutions’ GA is as follows:

Population: This offers numerous different approaches to the issue. The population size will not be directly related to the algorithm’s accuracy. The length of that individual depends on how many nodes are actually present in the network. If a node has a 1 instead of a 0, it is a CH, while a 0 means it is a member node. There is an arbitrary production of the initial population.

Fitness Function: This suggests adaptability. Based on the fitness function, the level of fitness for each person is determined. Regarding the current work, four different parameters are taken into account for this:

- The remaining energy;
- The number of CHs;
- The total intra-cluster communication distance;
- The total distance from the CHs to the base station.

As the number of CHs decreases, the distance between the CH and the BS will generally drop but the distance for intra-cluster communication will grow. The preceding two parameter values will be listed first.

This fitness function [11] has been described as

(1) $$Fitness=E+\left(N-CH\right)+\frac{IC}{N}+\frac{BSD}{N}$$

In this case, N stands for the quantity of already accessible network nodes. The entire distance between the CH and the BS has received additional emphasis, as can be seen in this function.

Selection: In order to create a new population, the approach selects individuals from the existing population. The main goal of employing this selection function for the GA was to give members who were more reproductively fit better chances. Some of the techniques used for the implementation of the process of selection are the random, rank, Boltzmann, tournament, and roulette wheel techniques.

Crossover: The probability of the crossover operation is determined by the rate of crossover, and it occurs between two distinct chromosomes. Both chromosomes that are segregated through the crossover site swap the sections as required.

Mutation: For each chromosome bit, a mutation operator is applied using the probability of mutation rate. The bit of 0 changes to 1 once a mutation is complete.

Table 1 shows a sample working for CH selection using GA.

### 3.2. Proposed CH Selection Using Dual Krill Herd Optimization Algorithm

An innovative optimization technique that aids in the resolution of extremely complicated problems is the KH. This is based on individual performance and belongs to the family of swarm intelligence. There are three different movements that are implemented, and these are again repeated in the KH. The solution that is the best is considered by the directions of the search. A krill position can be set in one of three ways: The effort exerted by the other krill;

- The foraging action;
- Physical diffusion.

The KH assumes a Lagrangian model as depicted here:

(2) $${dX}_{i}/dt=N_{i}+F_{i}+D_{i}$$

where $N_{i}$ represents the motion of the other krill, Fide notes the new seeking motion, and the physical distribution is $D_{i}$. According to Equation (2), the NP is represented by the variables *i* = 1, 2…, and it represents the population’s size.

The initial motion has a target that is local and has a repulsive impact that determines the motion’s direction, i. Equation (3) has been given as follows for the krill *i*…*N^max^*:

(3) $$N_{i}^{new}=N^{max}\alpha_{i}+\omega_{n}N_{i}^{old}$$

Its maximum attempted speed is indicated by *N^max^*, *ω_n_* is the weight of the inertia, and *N^old^* is the final motion.

The second motion will be determined by food location and earlier experience. In the case of the krill, it may be defined as in the following equations:

(4) $$F_{i}=V_{f}\beta_{i}+\omega_{f}F_{i}^{old}$$

(5) $$\beta_{i}=\beta_{i}^{pod}+\beta_{i}^{desr}$$

where *V_f_* denotes it looking for speed, *ω_f_* is the inertia weight for the second motion, and *F^old^* depicts the final motion.

The third motion is an unpredictable process with two distinct components, such as the highest diffusion speed and an unpredictable directional vector. Equation (6) is specified as follows:

(6) $$D_{i}=D^{max}S$$

where *D^max^* denotes the maximum speed flow; a random vector is denoted by δ.

Using three movements, the krill position from t to *t* + Δ*t* is represented as per Equation (7):

(7) $$X_{i}(t+\Delta t)=X_{i}(t)+\Delta t_{dt}{dX}_{i}$$

d(*n*_2_, CH_*n*+1_) = min {d(n_o_, CH_*n*_)} (8)

Other krill have an impact on the movement of the KH. Until a pausing condition is met, physical dissemination and foraging will continue for a number of these generations. Inter-cluster communication and intra-cluster communication are two different sorts of communication situations that can occur in a WSN. The work consists of a single-hop approach. Clustering was performed to improve intra-cluster communication and select an appropriate cluster representative from each round of nodes. Data obtained from different member nodes were aggregated at the level of the CH and forwarded to the BS. Through the use of this method, less energy was consumed. Yet, there may be a problem in that the CH is a stationary node and will eventually lose energy.

Therefore, for each round, there is a need to assign a new node to CH. There is a decision made to choose a node that is well suited, and this is taken up by the KH. The energy of the node and that of its separation nodes that are not CH members are used to select a new CH in each round. For operating the protocols of clustering, there are four different phases and two stages. The four phases are as follows: (1) selecting the CH; (2) formation of the clusters; (3) data aggregation; and (4) data communication. Two stages used are the setup state steady-stalemate stage. In this single setup phase, a sensor will transmit another location and its remaining data energy.

Thus, average energy is measured by the BS for every round. CH will be selected for that round based on the highest average energy, provided it is a capable node. This methodology is also further implemented to identify the K number for the fittest CHs. This brings down the cost of the function.

(9) $$f_{2}=\frac{\sum_{i=1}^{N}\;E\left(n_{i}\right)}{\sum_{i=1}\;E\left(C_{k}\right)}$$

(10) $$f_{3}=m\times f\left(x_{1}\right)=d_{pka}e^{\frac{51}{5}-55}$$

where f_1_ denotes the Euclidean distance average maximum among nodes of their related CH, and C_s_ indicates the actual nodes that best fit within the krill cluster C_k_. The function f_2_ is used to depict the relationship between the total and starting energy of the nodes En_3_, i = 1, 2…, N, found in the network and the total and presently available energy for the CH candidates in their actual round. β denotes the personally chosen constant which is used for weighing the contribution of every sub-objective. The fitness function has the distinct objective of bringing down the into-cluster separation measured between nodes and their CHs. This was quantified by f_1_:f_2_, which measures the effectiveness of energy found in the quantified network. According to the cost function definition, smaller values for f_1_ and f_x_ will mean the cluster is ideal and has the optimum number of nodes. This also means the cluster has the required energy to perform all tasks that are connected to the CH. The function f_s_ considers path delay, average energy, and successfully transferred data.

Step 1. Set the S krill for holding K of the CHs randomly designated among the CH candidates that are suitable.

Step 2. Calculation of the path cost function for every krill

i. For every node n′ = 1, 2, …, N,

Figure out the distance d (n_i_, CH_μ, ω_) between the node n_i_ and all the CHs^CH^p.

Delegate the node *E*^n^, to the CHCH_μ_ where in

(11) $$d\left(n_{2},{CH}_{n+1}\right)=\underset{w=1,\alpha }{min}\;\{(d_{o},{CH}_{n})\}$$

ii. Now estimate the cost function with equality.

Step 3. Find the perfect krill for each one and further identify the best-positioned krill. 

Step 4. Update the individual position in a search space.


dXi = deita, ×(N(i) + F(i) + D(i))
(12)

X(i) − X(i) + dU
(13)

Step 5. Repeat the steps 2 to 4 until the maximum iteration number is met.

**Figure 1:**
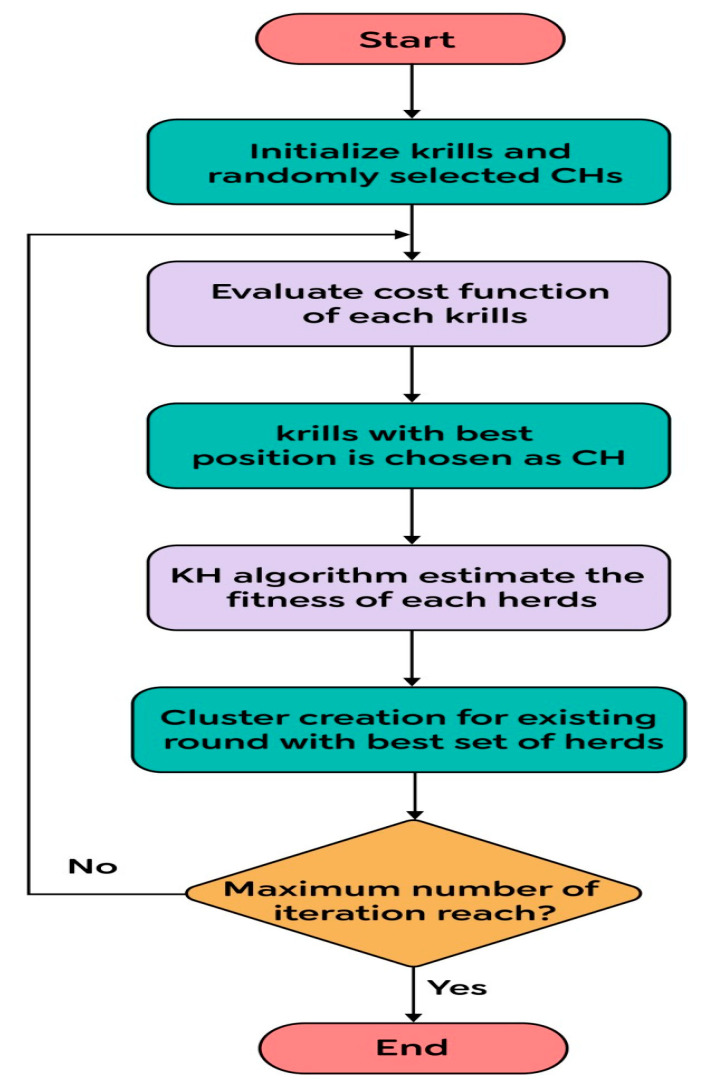
Flowchart for the proposed algorithm’s CH selection.

Information that consists of separate 𝔻 values for every CH to each and every node is communicated to the sensor field as soon as an optimal cluster combination is obtained by the BS. Clustering algorithms along with their process accomplished by incorporating KH into a WSN are depicted in Figure 1.

### 3.3. Dual-Cluster-Head Selection

The modified krill herd optimization approach selects master cluster heads instead of cluster heads. The three components of the coordinate axis in x, y, and z are used to identify the node’s position because the deployment environment is three-dimensional and underwater.

(14) $$X_{ix}(n+1)=X_{ix}(n)+[F_{i}^{new}+N_{i}^{new}+D_{i}^{new}]\cdot t$$

(15) $$X_{iy}(n+1)=X_{iy}(n)+[F_{i}^{new}+N_{i}^{new}+D_{i}^{new}]\cdot t$$

(16) $$X_{iz}(n+1)=X_{iz}(n)+[F_{i}^{new}+N_{i}^{new}+D_{i}^{new}]\cdot t$$

Due to the discontinuous distribution of the nodes in the water, it is impossible to precisely map the estimated value of the aforementioned formula onto the location of the real node. The cluster node position is therefore modified as follows:

Where pk denotes the position that is closest to the actual circumstance, *p_ix_*, *p_iy_*, and *p_iz_* are the exact values of the cluster’s components of x, y, and z, respectively, and *x_i_*(*n*) is the adjusted node position.

The implementation of a dynamic layered dual-cluster routing method in UWSNs based on krill herd optimization can now be said to have been accomplished with assurance. A list of these actions is as follows:

Step 1: Initializing the krill. It is necessary to establish each distinct random location in 3D space before modifying the position and projecting it onto the distribution of nodes in the water. 

Step 2: Determining the fitness value. Within the clusters, the krill individual extremum and the highest adaption values are determined to determine the krill’s present position. The global extremum of the krill swarm is where the krill are.

Step 3: Observe a change and relocate.

Step 4: Updates are made to the local and global extremums, and the updated adaption value is determined.

Step 5: In order to avoid exceeding the maximum number of repeats, steps 3 and 4 should be repeated.

Step 6: The master cluster head is chosen to be the global extreme.

Step 7: To eliminate the vice-cluster head, repeat the previous procedure using the value function on the vice-cluster head.

(17) $$p_{i}=\sqrt{{\left(p_{ix}\right)}^{2}+{\left(p_{iy}\right)}^{2}+{\left(p_{iz}\right)}^{2}}$$

(18) $$p_{k}=m*\left(p_{1},p_{2},\ldots ,p_{n-1},p_{n}\right)x_{id}(n)\approx p_{k}$$

### 3.4. Single- and Multi-Hop Transmission

Data are often transmitted by the vice-cluster head over one or more hops from the primary cluster head to the sink node. The vice-cluster head broadcasts its ID number, remaining energy, and other information initially. In the event that A chooses to be the vice-cluster head and receives the message from B, B delivers L bits of data to the sink node. Calculating the energy consumption model requires Equation (3). To ensure the power consumption and communication overhead of the next hop node, the weight of the sub-cluster head is established.

**Figure 2:**
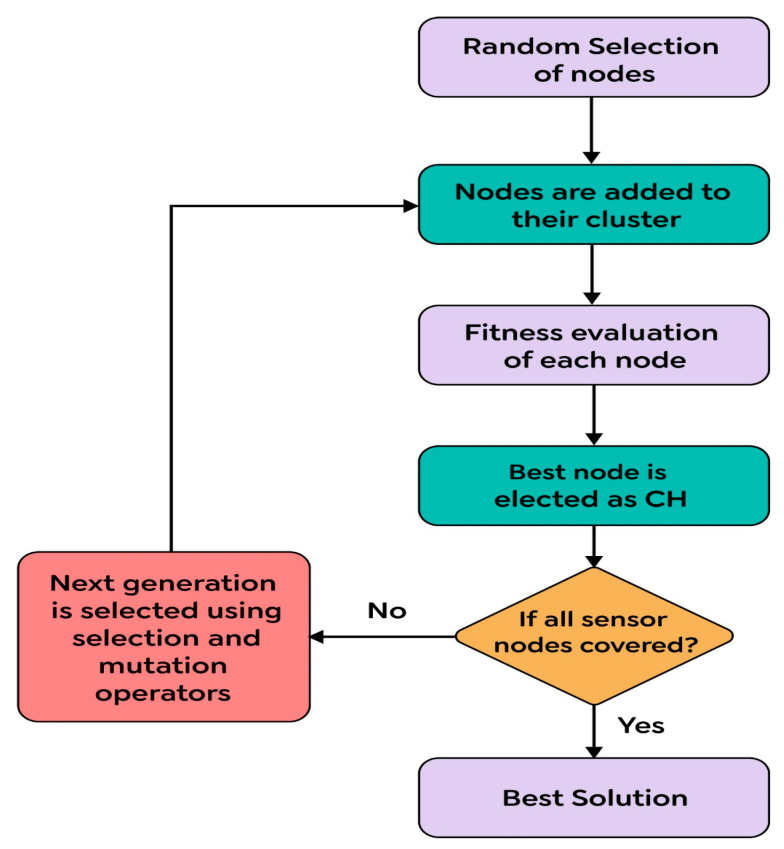
Optimal solution finding.

The actual situation determines the value of ∂. If W (i) is higher than the maximum, the next hop node is determined to be the vice-cluster head. The data are then sent directly from the vice-cluster head to the sink node. Figure 2 shows a flowchart for optimal solution finding.

### 3.5. Strategy of Data Aggregation and Communication

In the proposed enhanced data selection with K-means hybrid (EDS-KH) method, the data aggregation section involves employing a data aggregation algorithm, specifying aggregation rules, and implementing data fusion and compression techniques within each cluster to efficiently combine and process sensor data. Additionally, data communication is facilitated through a communication protocol between cluster heads and the base station, utilizing a routing protocol for optimal data transmission, considering quality of service (QoS) requirements. The data packet format is defined, and energy-aware communication strategies are incorporated to optimize energy consumption. To address data skewness, EDS-KH incorporates load balancing and re-clustering strategies to ensure an even distribution of data processing tasks and adaptive cluster head roles, enhancing the overall network’s efficiency and reliability in handling real-world data scenarios.

## 4. Comparison of Results

**Figure 3:**
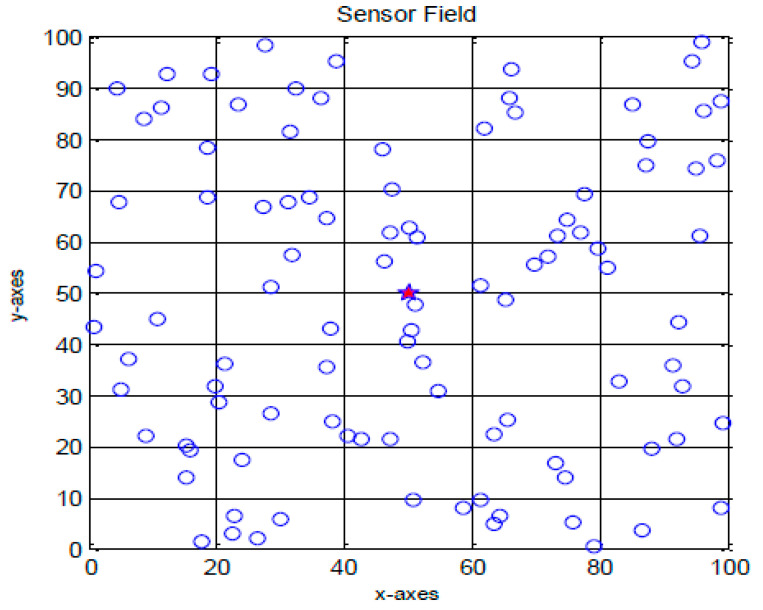
Sensor field used for experiment.

**Figure 4:**
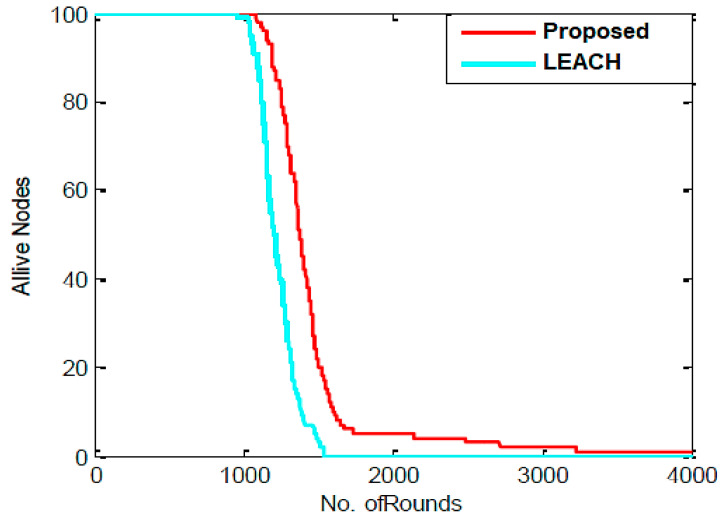
Comparison of live nodes in each round.

As shown in Figure 3, the proposed work involves randomly placing sensor nodes over a 100 m × 100 m region. The outcomes of the proposed algorithm are described in this section. Network lifespan and packets transmitted to BS are selected as aspects of performance to verify the proposed technique. The parameter settings for the proposed algorithm are shown in Table 2. Figure 4 depicts the experimental sensor field, in contrast. In the sensor field mentioned above, the dispersion of all sensor nodes is uniform, and it is assumed that the BS is situated within the sensor field. The MATLAB (v 2022) environment is used to implement the suggested procedure. The network lifespan parameter is determined by the ratio of active and inactive nodes. The proposed algorithm’s output is contrasted with that of the LEACH algorithm. The statistics for the network lifespan parameter are shown in Table 3. This table demonstrates that the initial sensor node in the LEACH algorithm dies after 1100 rounds since all of its energy was spent for transmission and data collection, that half of the sensor nodes die after 1265 rounds, and that there are no living nodes in the sensor field after 1570 rounds. After 1150 rounds in the proposed process, the first node is rendered useless because all of its energy was expended in data transmission and gathering. After 1340 rounds, the other half of the sensor nodes likewise perish, and after 3880 rounds, there are only 10 nodes in the sensor field that are still alive. It follows that the PSO algorithm’s use in LEACH increases the network’s lifespan and reduces the amount of energy used by nodes.

**Figure 5:**
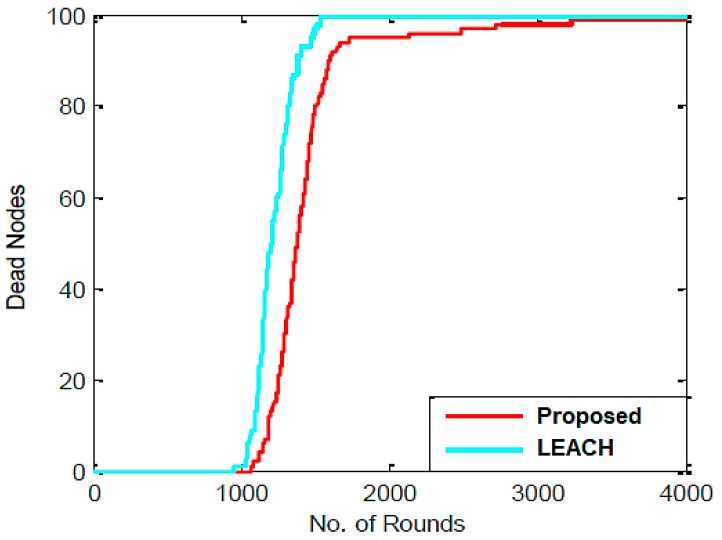
Comparison of dead nodes in each round.

Figure 5 illustrates how the LEACH and proposed algorithms compared in the amounts of living and dead nodes for each cycle. It is quite evident that the LEACH and proposed algorithms operate very differently from one another. All nodes in the LEACH and proposed algorithms die after 1570 and 3880 rounds, respectively, although it is noticeable that there is a considerable variation in the nodes’ death rate.

**Figure 6:**
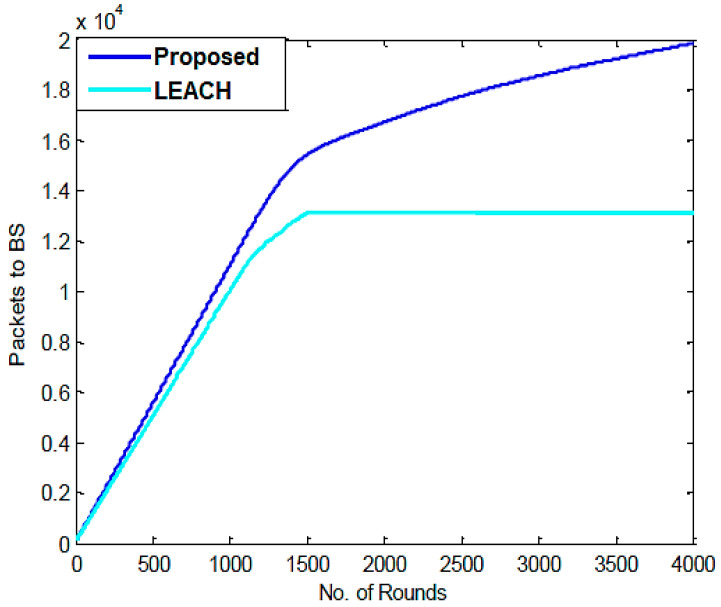
In each round, a comparison of the packets transferred from CHs to BS is made.

The information about packets transmitted to the CHs and to the BS for the LEACH and proposed algorithms after 500, 1500, 2500, 3500, and 4000 rounds is shown in Table 4. It has been noted that whereas the proposed algorithm sends out 20,000 packets, the LEACH protocol sends out 13,000. As the PSO algorithm was incorporated into the LEACH protocol, it appears that the number of packets sent has gradually increased. Figure 6 compares the packets transmitted to BS for each round using the LEACH and proposed protocols, demonstrating the gradual improvement in the LEACH protocol’s performance.

**Figure 7:**
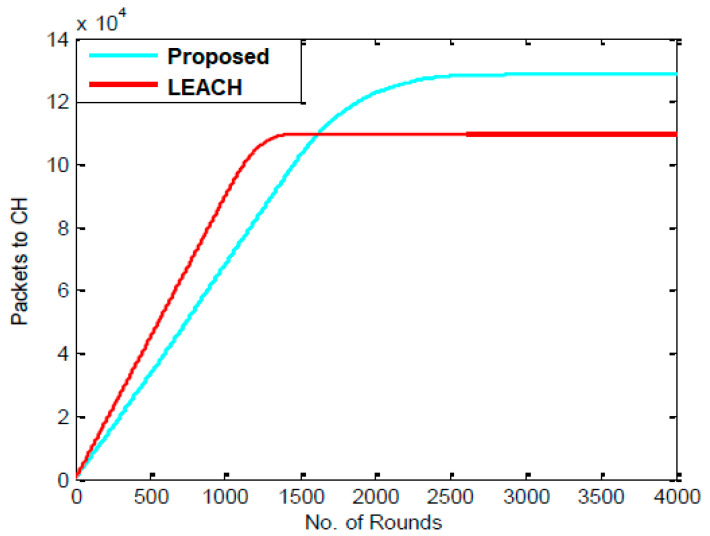
Comparison of packets sent to CHs from nodes.

Table 5 displays the information about packets transmitted from each node to CHs after 1000, 2000, 3000, and 4000 rounds for the LEACH and proposed algorithms. It has been noted that whereas the proposed protocol sends 130,000 packets, the LEACH algorithm sends 110,000 packets. Since the PSO algorithm was incorporated into the LEACH protocol, it appears that the number of packets transmitted has been increasing progressively. Figure 7 compares the packets transmitted to CHs for the LEACH and proposed protocols for each round, and it indicates that the LEACH protocols perform significantly better.

Figure 7 compares the number of packets transmitted to CHs during each round for the LEACH and proposed protocols, and it indicates that the LEACH protocols perform significantly better.

Lastly, it can be said that the LEACH protocol uses more energy than the proposed protocol nodes, which directly contributes to a longer network lifetime. The quantity of packets transmitted to BS and CHs is likewise steadily rising, confirming the impact of the suggested CH selection mechanism.

**Figure 8:**
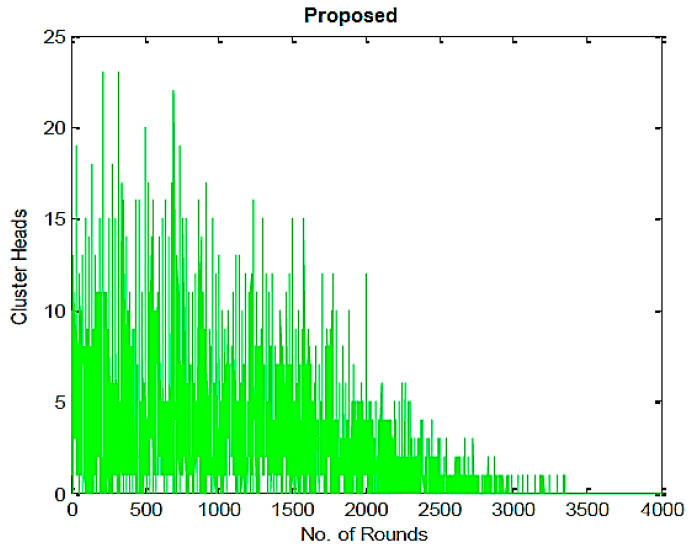
Packets transmitted to and from CHs by nodes, compared with the proposed algorithm.

**Figure 9:**
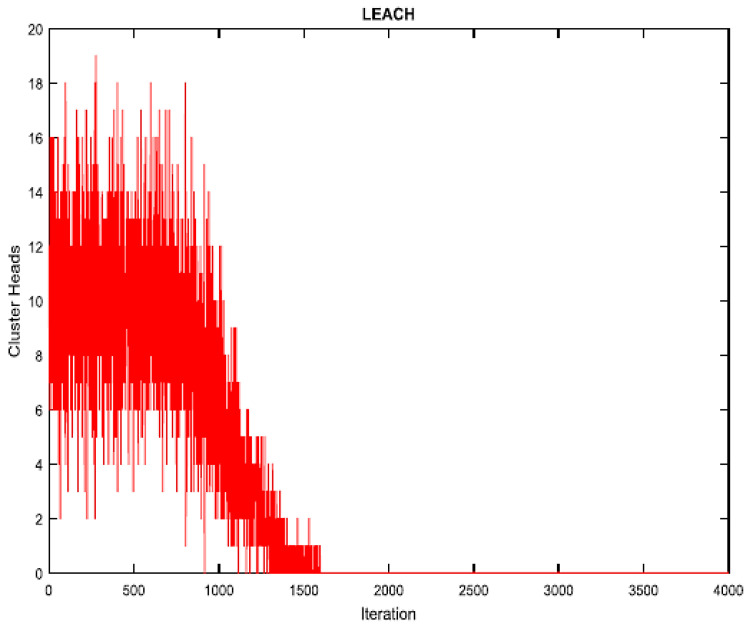
Number of CHs produced during the LEACH algorithm’s execution.

Figure 8 and Figure 9 provide the total amount of cluster heads produced by the proposed and LEACH algorithms in each iteration. It can be seen that the proposed method generates fewer CHs than the LEACH algorithm. Because of their higher stability compared to those of the LEACH algorithm, these CHs are significantly more energy-efficient.

**Figure 10:**
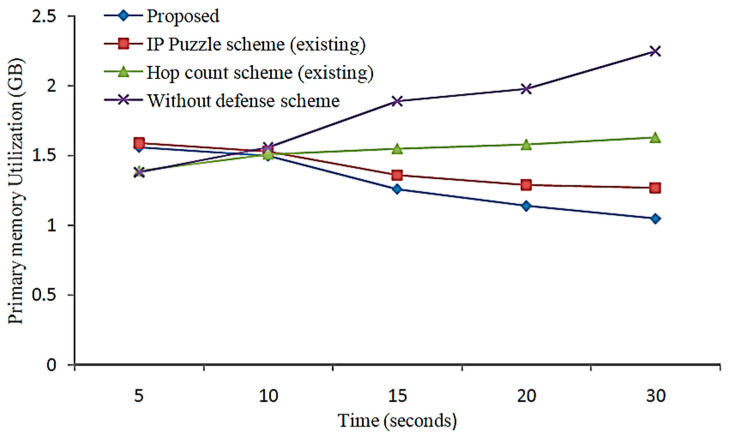
Connected living nodes with their current round counts.

Figure 10 indicates the comparison of cluster heads present, as well as the fitness which is used for four values of iteration. As can be seen from the figure, the percentage difference in the fitness value for each value of iteration is almost the same. Hence, even for larger values of iterations, the fitness function does not change its value. While the quantification of the existing number of heads clustered is less, the fitness quantity is maximum for all values of iteration. So, as the fitness function decreases, the durability of a network over time decreases.

### 4.1. Comparison Study

With the use of Python 3.6 and its supplementary libraries—including Matplotlib, NumPy, and Network—the effectiveness of the suggested plan is contrasted with that of MBABCOA, KHOGACP, and GSACP. The homogeneous and heterogeneous setups are used to test the proposed system and the three reference schemes (GSACP, MBABCOA, and KHOGACP). With a uniform configuration, the whole nodes of the sensors are the part of the network having the same amount of energy to start with. While the sensor nodes in a heterogeneous configuration have varied amounts of available power, the setup has several advantages. For an accurate comparison of energy efficiency and network longevity, the simulation was carried out in conditions analogous to those of the experiments. There are one thousand sensor nodes in the simulation environment, and they are spread out throughout a 400 × 400 m terrain area so that anyone may evaluate the suggested method in a realistic setting. It is common practice to assume that the network’s base station is situated in its geographic epicenter. Table 6 displays the simulation settings used to test the proposed scheme in comparison to the baseline KHOGACP, GSACP, and MBABCOA approaches to choosing heads that are currently in use.

**Figure 11:**
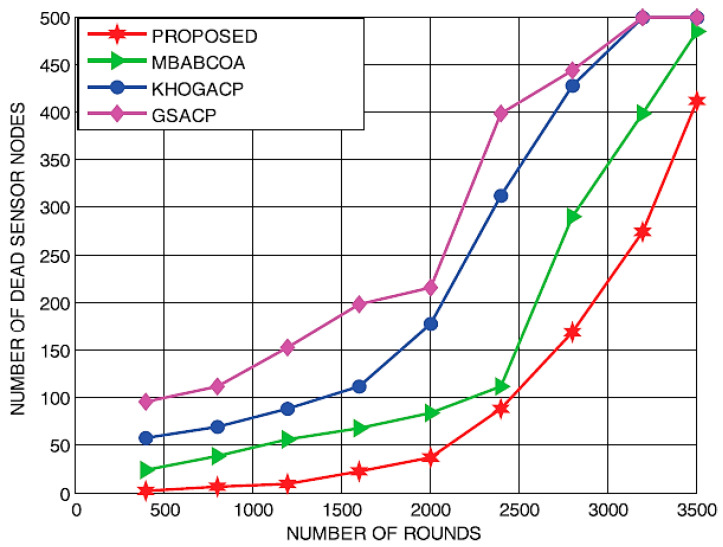
Nodes that have died, accompanied by a count of rounds.

During the initial stage of the investigation, it was discovered that the suggested scheme had greater possibility than the baseline GSACP, KHOGACP, and MBABCOA systems that differ in terms of the number of cycles, the quantity of remaining energy, the number of living nodes, and the number of dead nodes. Figure 10 and Figure 11 represent the implementation readiness evaluation of the number of times used to count the number of dead nodes and the number of surviving nodes in total, respectively. With further iterations, more sensor nodes are expected to remain functional. At the same time, the planned system at the 3500th round is thought to have kept alive 93 sensor nodes. Yet, in rounds 2990, 3280, and 3500, respectively, the total number of nodes that are maintained alive by the basic GSACP, KHOGACP, and MBABCOA systems is nearly nil.

During the process of selecting cluster heads, an ideally enhanced force FOA method led to an increase in the rate of exploitation that reached the level that had been projected. Therefore, it has been determined that the proposed scheme is as effective as the benchmarked schemes in managing the energy balance of the sensor nodes that are part of the network. As the total number of rounds keeps growing, however, the figures show that the proportion of dead sensor nodes grows steadily. At the same time, 96 nodes are supposed to represent the number of nodes that are operational and functional on the 3500th round if the proposal is put into action. After 3250, 3280, and 3500 rounds in the benchmarked GSACP, KHOGACP, and MBABCOA systems, respectively, most of the nodes have already died off. The proposed scheme is clarified to sustain the sensor’s life span nodes in comparison to the benchmarked schemes because it included a rapid and dependable good compromise between exploration and extraction, which helped to prevent the very least suited nodes from becoming selected as the heads of clusters.

**Figure 12:**
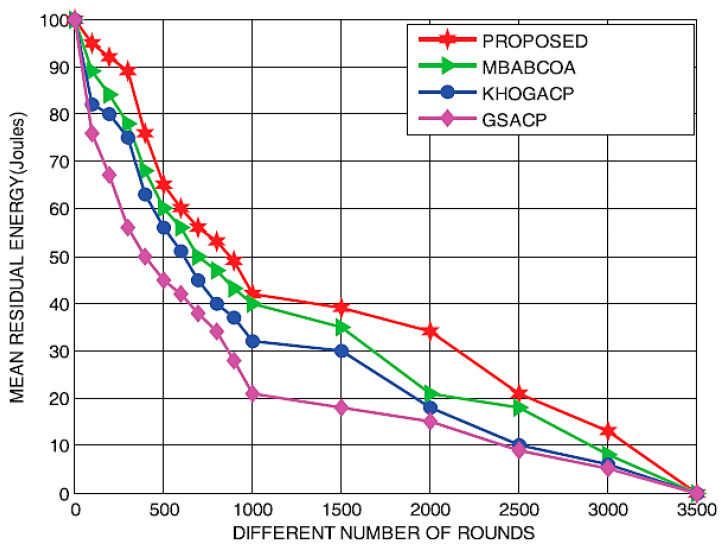
Energy remaining after a certain number of rounds.

**Figure 13:**
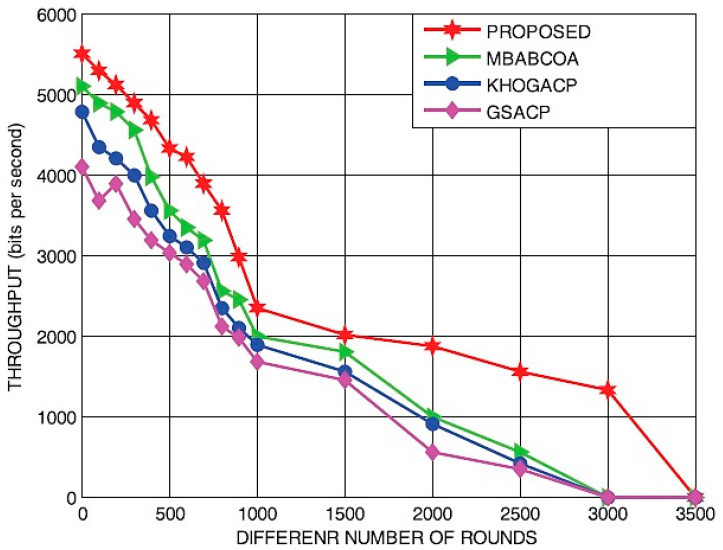
Productivity in relation to iterations.

Figure 12 and Figure 13 depict residual energy and output as a function of increasing round counts. Our results showed that with each successive round, leftover power for all four schemes (the baseline MBABCOA, KHOGACP, and GSACP and the suggested scheme) decreased. The mean leftover power of the proposed model has been shown to be similar to that of the benchmarked schemes as the number of cycles increases. There is little doubt in anyone’s mind that the proposed technique excels since it does not require a constant selection of cluster heads. Increasing the total amount of rounds to be in line with the most prominent models has also been verified to significantly improve the throughput of the intended scheme. Exploration rate and clustering must be well balanced for the proposed method to attain its remarkable performance.

**Figure 14:**
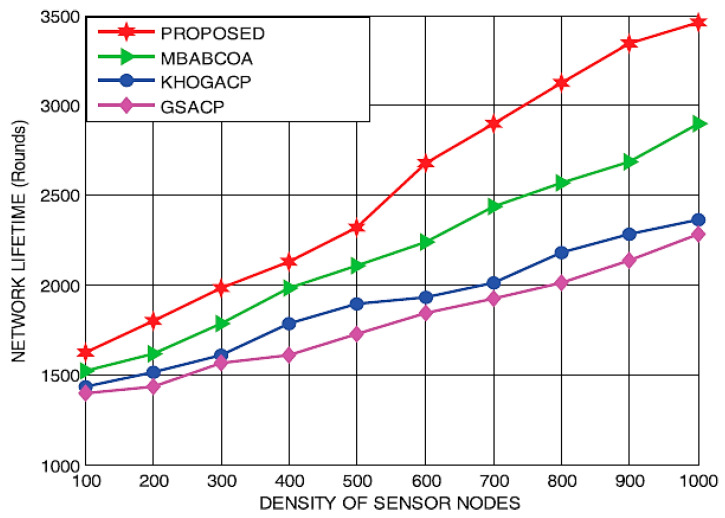
Comparison of network lifetimes for low, medium, and high sensor node density.

**Figure 15:**
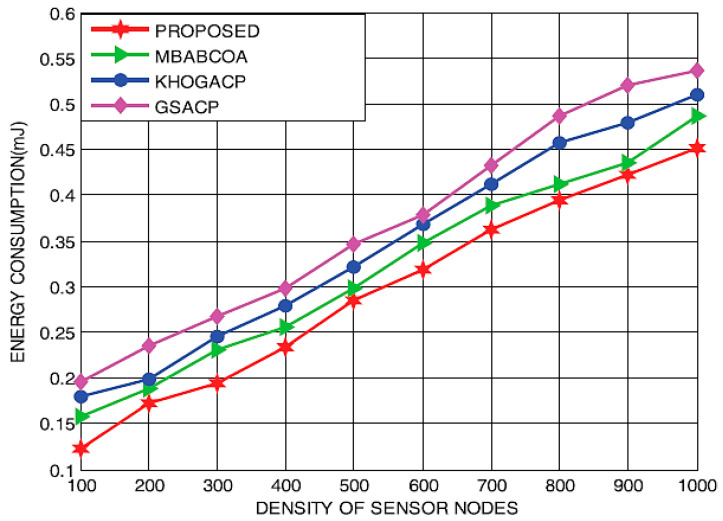
Impact of sensor density on energy use.

In the second part of the study, the suggested scheme is contrasted with the starting point of the KHOGACP, GSACP, and MBABCOA systems, and their performance for the different densities of sensor nodes is measured in terms of lifespan, utilized energy, throughput, and packet delivery rate. Figure 14 and Figure 15 display the lives and energy requirements of the suggested and baseline GSACP, KHOGACP, and MBABCOA systems with varying network sensor node densities. As it is impossible for sensor nodes to become cluster leaders along with the loss of energy eradicated, the proposed network lifespan grows in tandem with the quantification of sensor nodes. Furthermore, the suggested method considerably reduces the power usage of the sensor nodes by removing the potential for any limits to be reached during the exploration and exploitation stages. The suggested approach is expected to make the network last longer by 19.21%, 17.54%, and 14.29% compared to the starting point of the KHOGACP, GSACP, and MBABCOA systems which depend on density sensor node distributions. In addition, the suggested method reduces energy usage by 18.61%, 15.36%, and 12.82% compared to GSACP, KHOGACP, and, MBABCOA, respectively, for three different sensor node densities.

**Figure 16:**
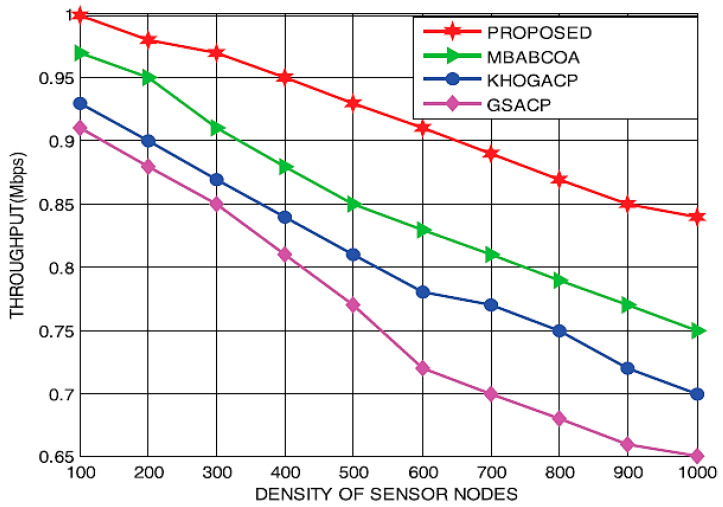
Processing capacity at varying sensor densities.

Figure 15 and Figure 16 display the delivery time and throughput efficiency of the proposed network at varying sensor node densities, respectively. A huge amount of energy is lost in the network, and the rate of transmission and rate of packet delivery decrease as the number of sensor nodes grows, even when a large number of packets are being sent out at once. The suggested method, which can be used in networks with varying sensor node densities, achieves greater delivery time and throughput efficiency rates than the reference implementations because it selects only the most reliable and energy-efficient indicator nodes to act as cluster leaders for data collection. As a result, the proposed scheme’s throughput is 17.64% higher than that of the baseline GSACP, KHOGACP, and MBABCOA systems, all of which make use of a network with differing densities of sensor nodes. The suggested strategy reduces the packet delivery rate by 16.71%, 14.83%, and 11.283% in contrast to the starting point of the KHOGACP, GSACP, and MBABCOA plans with differing sensor node densities.

**Figure 17:**
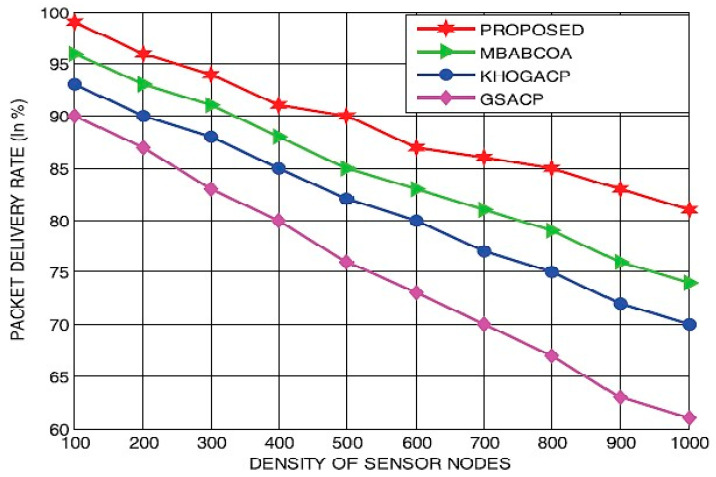
Different sensor node densities’ effects on the delivery rate of packets.

The research concludes with a look at what would happen if all of the nodes in the network suddenly stopped working, as well as what would happen if half of them died and the other half stopped working. In the suggested strategy, the first sensor node is doomed to fail after 112 rounds; by round 468, 50 percent of the sensor nodes will have perished, and the final sensor node will die at round 512. Results from a study of the proposed system’s protocols, conducted with network existence scenarios in mind, are shown in Figure 17. The suggested success is anticipated to increase the life of the network by 18.22%, averaging 21.39% when compared to the 20.18% and 22.94% gains made by the GSA-CRS, CHS-OCHS, and KHA-OCHS, IMBOA-ABC-CHS, starting point techniques.

**Figure 18:**
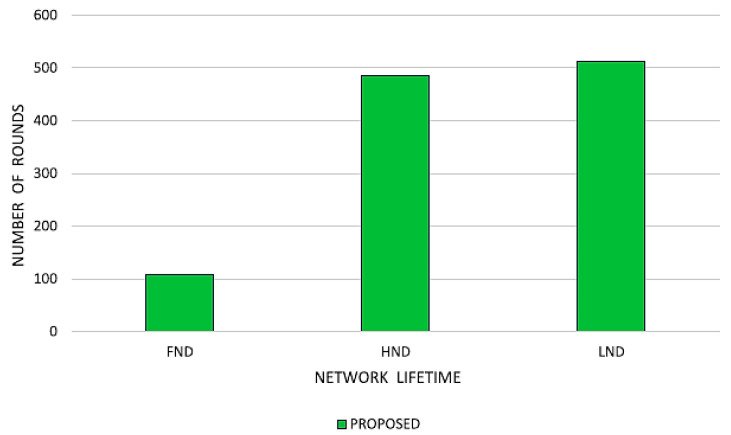
Future of the proposed network scheme.

**Figure 19:**
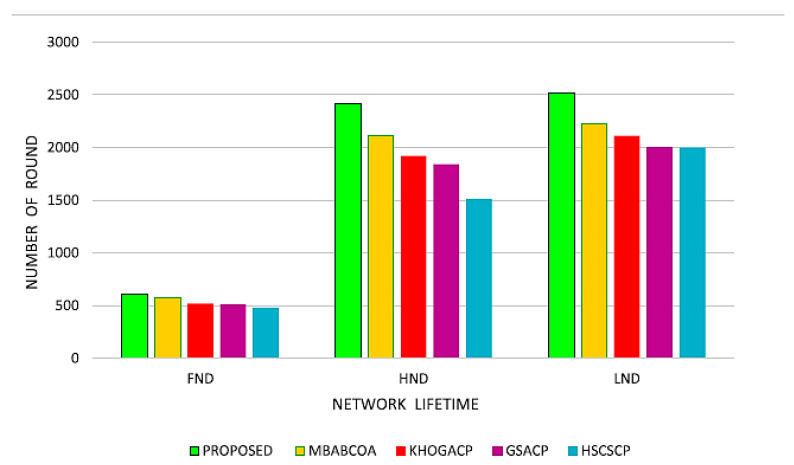
The suggested scheme’s expected lifespan in a network.

The benchmarked protocols evaluated with a homogeneous and heterogeneous network design, as well as the network security (sensor nodes alive), are shown in Table 7 and Table 8. In contrast to the GSACP, KHOGACP, HSCSCP, and MBABCOA baseline approaches, the proposed design improves stabilization time by 21.36 percent, 16.21 percent, 14.84 percent, and 12.8 percent, respectively, as shown in Figure 7 and Figure 8. Increases of 18.21%, 17.38%, 13.96%, and 11.34% are also proposed in the program. The stability time under a heterogeneous setup was thought to be lengthened by using KHOGACP, GSACP, and HSCSCP as opposed to the baseline method of MBABCOA. The overall goal of the present system proposal is to increase network longevity by 3.48 percent, 4.98 percent, 5.96 percent, and 6.36 percent. It was determined that the suggested method has the same minimal duration of unstable life as the CH selection strategies, confirming that its primary purpose is to increase stable life. Table 9 and Table 10 illustrate the selected active nodes during the identical and heterogeneous setup rounds, each in the proposed procedure. Since dynamic high tide force is implemented to enhance exploitation and overcome the constraints on modern ABC in CHS, the proposed protocol has demonstrated greater efficiency in relation to homogeneous and heterogeneous setups and first-, second-, and third-order node failures (Figure 18 and Figure 19).

### 4.2. Discussion

The proposed enhanced data selection with K-means hybrid (EDS-KH) method is a novel clustering algorithm specifically designed to overcome the limitations of existing clustering algorithms in wireless sensor networks (WSNs). The method incorporates elements of K-means clustering and hybrid elephant herd optimization (EHO) to enhance the efficiency and adaptability of cluster head selection, data aggregation, and routing processes. More details on the EDS-KH method and how it addresses the limitations of existing clustering algorithms are as follows:

- Adaptive Cluster Head Selection: EDS-KH utilizes the K-means clustering algorithm to dynamically adapt the cluster head selection process based on changing network conditions. Unlike the traditional K-means method that requires a fixed number of clusters (K), EDS-KH adjusts K based on the network’s current state, leading to improved cluster formation. By utilizing adaptive cluster head selection, EDS-KH overcomes the limitation of fixed K in K-means and other clustering algorithms, which may lead to suboptimal cluster head choices and inefficient energy distribution.
- Energy Balancing: The EDS-KH method incorporates the hybrid elephant herd optimization (EHO) to achieve energy balance among sensor nodes. EHO mimics the social behavior of elephants, promoting efficient energy utilization and preventing hotspot issues, where some nodes deplete their energy faster than others. Through energy-balancing mechanisms, EDS-KH addresses the limitations of existing clustering algorithms that may fail to consider energy distribution among sensor nodes, resulting in premature battery depletion and reduced network lifetime.
- Handling Irregular Clusters and Varying Densities: EDS-KH leverages the benefits of both K-means and hybrid EHO to efficiently handle irregularly shaped clusters and varying cluster densities. K-means ensures accurate data partitioning, while EHO’s adaptive exploration strategies address the challenges posed by non-spherical and overlapping clusters with varying densities. By overcoming these challenges, EDS-KH surpasses the limitations of existing clustering algorithms, such as K-means, which may struggle to accommodate irregular cluster shapes and varying data densities.
- Improved Fault Tolerance and Network Robustness: The EDS-KH method incorporates enhanced elitism and mechanisms to prevent the selection of the cluster leader from the worst sensor node. This fosters improved fault tolerance and network robustness by ensuring that cluster heads are selected from relatively more stable and reliable nodes. By enhancing fault tolerance and robustness, EDS-KH addresses the limitations of existing algorithms that may not explicitly consider the selection of more stable cluster heads, potentially leading to network disruptions in the presence of node failures.

Thereby, the EDS-KH method offers a comprehensive approach to clustering in WSNs, addressing the limitations of existing algorithms by incorporating adaptive cluster head selection, energy balancing, handling irregular clusters and varying densities, and enhancing fault tolerance and network robustness. These enhancements make the EDS-KH method a promising solution for optimizing the performance and longevity of wireless sensor networks in various real-world applications.

In addition to the proposed enhanced data selection with K-means hybrid (EDS-KH) method, the authors are encouraged to consider adaptive topology management schemes for maintaining network connectivity in wireless sensor networks (WSNs). Adaptive topology management involves dynamically adjusting the network’s structure to respond to changing environmental conditions and node failures, ensuring continuous communication and data transmission. Techniques such as dynamic clustering, node reconfiguration, and mobile sink deployment can be incorporated to adaptively reorganize the network and maintain connectivity. By employing adaptive topologies, WSNs can effectively handle node mobility, topology changes, and network partitioning, improving data delivery, fault tolerance, and overall network performance. Integrating adaptive topology management with EDS-KH can further enhance the resilience and adaptability of a WSN, making it more suitable for complex and dynamic environments while extending its applications to mission-critical scenarios like disaster response and environmental monitoring.

## 5. Conclusions

In this paper, EDS-KHO is proposed as a reliable power convergence clustering methodology with shorter distances between nodes and less time between them. This is done to keep the expected level of a wireless sensor network’s average lifespan. Indications showed that KHO adds tidal force to its dual mechanism to improve how it works and eliminate the problem of postponed convergence. In this incorporation of the customized EDS-KHO, positions that have not been updated with the new positions made by the trooper bee shift in the process. As part of their work, bees are used in a procedure; in addition to this, there is a fresh search formula to increase the chance of accurately predicting desirable placements, primarily by swapping out some less favorable sites during the viewing phase for possible new ones. The results of the suggested scheme’s simulation supported the stabilization period for a relatively homogeneous setup of the available approaches, GSACP, HSCSCP KHOGACP, and MBABCOA by 22.37, 13.22, 15.85, and 12.36 percent, respectively. These percentages are in accordance with the suggested scheme. When compared to benchmark systems, the stabilization time is also thought to increase by 18.21%, 17.38%, 13.96%, and 11.34. Spotted baboon optimization and the linear search clustering procedure will likely be developed soon so that they can be compared to the proposed system.

The proposed enhanced data selection with K-means hybrid (EDS-KH) method holds considerable promise for enhancing wireless sensor networks (WSNs); however, several potential limitations should be addressed in future research. These include investigating its scalability for large-scale WSNs and fine-tuning parameters for optimal performance. In future research, the scalability for large-scale WSNs and fine-tuning parameters for optimal performance in the proposed enhanced data selection with K-means hybrid (EDS-KH) method can be addressed through approaches such as distributed and parallel algorithms, hierarchical clustering, automated parameter tuning using machine learning techniques, sensitivity analysis, extensive simulation and real-world deployment, and fostering collaborative research efforts. By exploring these strategies, researchers can enhance the efficiency, adaptability, and performance of EDS-KH, making it a more practical and effective solution for diverse WSN applications and challenging network conditions.

## Figures and Tables

**Table 1:** Sample working for CH selection using GA.

Original CHs	C1s	C2s	C3s	C4s	C5s	C6s	C7s	C8s	C9s	C10s	Range
Node 1	1	0	0	1	0	1	1	1	1	0	0.86
Node 2	0	1	1	0	1	1	1	0	1	0	0.250
Node 3	1	1	1	1	0	0	1	1	1	0	0.804
Node 4	1	1	0	1	0	1	1	1	1	1	0.135
Node 5	1	0	0	1	1	1	0	1	1	1	0.187
Node 6	0	0	0	1	1	1	0	1	0	0	0.338
Node 7	1	1	1	0	1	0	0	0	0	0	0.483
Node 8	0	0	0	0	0	0	1	0	0	1	0.802
Node 9	0	1	1	0	0	1	1	0	1	0	0.555
Node 10	1	0	0	0	1	0	0	1	0	1	0.789
Node 11	0	1	1	1	0	1	0	1	1	0	0.474
Node 12	1	1	0	0	1	0	1	0	0	1	0.519
Node 13	0	0	1	1	1	0	0	0	1	0	0.250
Node 14	1	0	1	0	1	1	1	1	0	1	0.626
Node 15	1	1	1	1	0	1	0	0	1	1	0.365
Node 16	0	1	0	0	0	0	1	1	0	1	0.546
Node 17	0	0	0	1	1	0	0	0	1	0	0.876
Node 18	0	0	0	0	0	1	0	1	0	0	0.370
Node 19	1	1	1	1	0	1	0	0	1	1	0.788
Node 20	0	1	0	0	1	0	0	0	0	0	0.434
Node 21	1	0	1	1	0	0	1	1	1	1	0.743
Node 22	0	0	0	1	1	1	1	1	0	0	0.113
Node 23	1	1	1	1	1	0	1	0	0	0	0.470
Node 24	1	0	0	1	0	1	0	1	1	0	0.542
Node 25	0	1	1	0	1	0	1	0	1	0	0.889
Node 26	1	0	0	0	1	1	1	0	1	1	0.140
Node 27	0	1	0	1	0	0	1	0	1	1	0.780
Node 28	0	0	0	1	0	0	1	1	0	0	0.835
Node 29	1	1	1	0	1	1	0	1	0	0	0.30
Node 30	1	0	0	0	0	1	0	1	0	0	0.34

**Table 2:** Setting the proposed algorithm’s parameters.

Parameters	Factors	Limits
Size of network	Field of net	(100 × 100) m
No. of nodes in the field	No. of network	100
Original power of node J	Eo	0.5 Joule
Power of transmission (nJ/bit)	ETX	50 nano joule/bits
Power of reception (nJ/bits)	ERX	50 nano joule/bits
Weight of inertia	Efs	10 P joule/bits
Power of data aggregation (nJ)	Winit	0.45
Power of amplifier J	EDA	5 nano joule/msg bits
Size of msg	Emp	0.0013 p joule/bit
CH probabilities	Size of Msg	40,000 bits
No. of repetition	No. of repetitions in max	0.10
Factor of acceleration	C1 = C2	4000
Particle velocity	Vmax	2

**Table 3:** Number of dead nodes over the network’s lifespan.

Techniques	No. of Repetitions		
	1st Death in Network	½ Dead in Network	Final Death of Network
Proposed	1151	1341	3881
LEACH	1101	1266	1571

**Table 4:** Base station received packets (in proposed 10 nodes remaining after 4000 rounds).

No. of Repetition Nodes (S)	No. of Sent Packets	
	LEACH	Proposed
500 s	5001	7001
1500 s	11,001	13,501
2500 s	13,001	17,061
3500 s	13,001	18,601
4000 s	13,001	20,001

**Table 5:** Packets sent to cluster head.

No. of Repetition Nodes (S)	No. of Sent Packets	
	LEACH	Proposed
1000 s	48,001	56,001
2000 s	105,001	122,501
3000 s	110,001	130,001
4000 s	110,001	130,001

**Table 6:** Factors of simulation.

Factors	Limits
No. of network sensor	1000
Network size	400 × 400 sq
BS position	(50, 150) m
No. of implementation rounds	3500 J
Original power of homogeneous setup	1 J
Original power of heterogeneous setup	Rand (0.5, 1) J
Length of Msg from Src to Des	2800 bits
Length of Pac from Src to Des	6400 bits
CHs probabilities	5%
Power of data used	5 nJ/Bits

**Table 7:** Lifespan (in terms of iterations) of the proposed strategy with a homogeneous setup, including its stable and unstable phases.

Homogeneous Setup					
Periods	Proposed	MBABCOA	KHOGACP	GSACP	HSCSCP
Period of stability	2664	2416	2216	2206	2197
Period of instability	512	662	674	736	798
Period of lifetime	3255	3112	3006	2946	2842

**Table 8:** Duration (in rounds of network operation) of the proposed method with a heterogeneous configuration during its stable, unstable, and active phases.

Heterogeneous Setup					
Periods	Proposed	MBABCOA	KHOGACP	GSACP	HSCSCP
Period of stability	2171	2112	1982	1963	1683
Period of instability	172	268	342	497	558
Period of lifetime	2345	2142	2210	2201	2207

**Table 9:** The number of rounds required to ascertain which nodes are still functioning under a uniform configuration of the proposed protocol.

Homogeneous Setup					
Nodes Alive (%)	Proposed	MBABCOA	KHOGACP	GSACP	HSCSCP
**0**	3225	3199	3095	2985	2981
**10**	3197	3143	2977	2917	2764
**20**	3123	3079	2933	2854	2598
**30**	3049	2949	2855	2795	2498
**40**	3015	2925	2763	2833	2678
**50**	2913	2853	2819	2799	2876
**60**	2845	2783	2647	2657	2345
**70**	2758	2783	2620	2467	3467
**80**	2715	2732	2550	2567	2541
**90**	2895	2687	2647	2357	2322
**99**	2687	2599	2456	2465	243

**Table 10:** Nodes still functioning after a certain number of rounds of running the suggested protocol in a mixed environment.

Heterogeneous Setup					
Nodes Alive (%)	Proposed	MBABCOA	KHOGACP	GSACP	HSCSCP
**0**	3228	2247	2234	2116	2314
**10**	3198	2239	2178	2123	2356
**20**	3124	2456	2167	2134	2245
**30**	3048	2378	2245	2178	2116
**40**	3013	2211	2345	2245	2456
**50**	2916	2234	2378	2236	2768
**60**	2847	2267	2367	2435	2876
**70**	2759	2456	2189	2478	2567
**80**	2715	2567	2341	2456	2667
**90**	2895	2478	2318	2678	2876
**99**	2684	2567	2451	2367	2765

## Data Availability

Not applicable.
